# Innovative Drug Delivery Systems for Regenerative Medicine

**DOI:** 10.3390/pharmaceutics16020295

**Published:** 2024-02-19

**Authors:** Elise Verron

**Affiliations:** 1 Chemical and Interdisciplinarity, Synthesis, Analysis, Modelisation, CEISAM UMR CNRS 6230, University of Nantes, 44300 Nantes, France; elise.verron@univ-nantes.fr; 2 Department of Elaboration and Evaluation of Drugs, Faculty of Pharmaceutical and Biological Sciences, University of Nantes, 44000 Nantes, France; 3 Center of Mineral Elements Dosage (CDEM), Faculty of Pharmaceutical and Biological Sciences, University of Nantes, 44000 Nantes, France

## 1. Introduction

In the past two decades, research on drug delivery systems has achieved significant advances. Initially, drug delivery systems were designed to provide a local release of drugs to improve their weak bioavailability and their tolerance as compared to a systemic administration [1]. However, they often displayed a short-term release not adapted for chronic disease. Today, the fine tuning of drug release, depending on the pharmacokinetic properties, is possible due to suitable systems [2]. In fact, these sophisticated systems are not only helping to target delivery to tissues or organs but also to comprehensively regulate the drug’s time and dose distribution in the body. As a consequence, they have revolutionized the field of tissue engineering and regenerative medicine.

Regenerative medicine applies the principles of engineering and life sciences to deal with the regeneration or replacement of damaged or diseased tissues and organs [3]. Due to the aging world population, multiple traumas, and diseases, the demand for organ replacements is increasing [4,5]. In order to promote tissue/organ repair or reconstruction, regenerative medicine needs to combine basic sciences including molecular and cellular biology and biomechanics with applied sciences such as polymer chemistry, nanotechnology, and bioinformatics [6]. Regeneration of a tissue/organ is a very complex process and often requires the combination of several strategies, such as the development of multifunctional scaffolds, the delivery of growth factors or other biochemical signals, cell and gene therapies, immunomodulation, and the use of external stimuli, i.e., electrical or magnetic pulses.

This Special Issue, entitled “Innovative Drug Delivery Systems for Regenerative Medicine”, aims to introduce the most advanced technologies, which contribute to the research and development in the field of regenerative medicine. This impressive collection of ten innovative works—six original research studies and four review papers—describes emerging advances in the development of smart systems suitable for local drug delivery, employing various strategies (i.e., localized delivery, targeted/triggered strategies) and highlighting their advantages in this research area.

To summarize, tissue engineering and regenerative medicine seek to treat patients with dysfunctional tissue, and the development of innovative biomaterial-based technologies is the subject of intensive research [7,8].

## 2. Overview of the Published Articles

The selected original research studies addressed various therapeutic applications to demonstrate that numerous tissues and organs are affected by these revolutionized smart systems. Driving and enhancing vascularization is essential for the repair and the regeneration of tissues consequent to severe lesions/damages. In their study, Gallo et al. (contribution 1) were interested in the essential amino acid L-lysine due to its involvement in proangiogenic, proregenerative, and anti-inflammatory mechanisms. They designed an optimized PLGA-based microparticulate system for maximizing L-lysine encapsulation efficiency and tuning its release kinetics. Another critical challenge in regenerative medicine concerns the design of implants for tissue transitions as observed, for example, in the rotator cuff in the shoulder with its direct osteo–tendinous junction (i.e., entheses). This is due to gradients in characteristics that need to be restored. The approach by Berten et al. (contribution 2) towards an optimized implant for entheses was based on electrospun fiber mats of poly(ε-caprolactone) (PCL) as a biodegradable scaffold material, loaded with chitosan/tripolyphosphate (CS/TPP) nanoparticles containing transforming growth factor-β3 (TGF-β3). The synthesis protocol was optimized to provide a controlled release of TGF-β3 resulting in an effective chondrogenic differentiation of human mesenchymal stromal cells. Furthermore, other categories of tissue regeneration were discussed in this Special Issue, for example, spinal cord injury treatment by Gonzalez et al. (contribution 3) through their promising polymers based on silk-elastin-like polymers, treatment of retinopathies due to the new long non-coding RNAs identified by Sharma et al. (contribution 4), and cardiac degeneration by Meligny et al. (contribution 5).

Finally, this Special Issue includes original articles dedicated to the development and validation of a synthesis method. For example, Rosiak et al. (contribution 6) developed a smart mathematical model to select the appropriate weight ratios of each component of the formula. Their model allowed improving the solubility and antioxidant activity of pterostilbene. Interestingly, they used a low-cost and green approach involving dry milling for carrying out the amorphization process. Moreover, Shirosaki et al. (contribution 7) conducted an experimental study to characterize and follow the loading and release of a drug from sophisticated capsules, whose mechanical strength and permeability need to be controlled.

This Special Issue also offers interesting reviews, which deeply explore the recent advancements in the spatiotemporal delivery of bioactive agents with optimal stability, activity, and tunable delivery for effective sustained disease management. These reviews have also been selected to cover different therapeutic approaches. In their review, Da et al. (contribution 8) described advancements in the pivotal components in delivery systems, including biomedical innovations, system fabrication methods, and loading strategies, which may improve the performance of delivery systems for better regenerative effects to treat lower genitourinary injuries. The generalist review written by Mansour et al. (contribution 9) provided an overview of different approaches used for drug delivery depending on the pharmacokinetic objectives that need to be reached. It included 3D-printing and electrospinning methods and innovative materials such as nanomaterials and extracellular vesicles. They concluded by providing interesting future directions. Lastly, the review by Guo et al. (contribution 10) focused on titanium (Ti) dental implants. Although Ti is the ideal material for dental implants with favorable biocompatibility and biomechanics, chemical corrosions arising from interaction with the surrounding tissues and fluids in oral cavity can challenge the integrity of Ti implants. Guo et al. explored smart solutions to design the next generation of therapeutic and corrosion-resistant dental implants from nanoscale surface modifications.

## 3. Conclusions

Regarding active compound delivery systems, the spatiotemporal control of the release profile represents a real challenge in terms of the optimal activity, stability, and safety and would offer personalized medicine. In response to these challenges, sophisticated delivery systems are emerging such as the ones based on nanotechnologies. Major mechanisms and critical factors affecting release profiles are being deeply studied through mathematical model contributions.

To summarize, this Special Issue highlights the significant progress, promising outlook, and futures challenges for regenerative medicine. These studies provide valuable insights into the development of efficient and effective medical treatments using sophisticated drug delivery systems.

As guest editor, I am very grateful and express my deep appreciation to all of the authors, who are experts in the field of regenerative medicine and responded to my invitation to share the findings of their high quality research, as well as for the critical assessments of their manuscripts.
